# Insights into the Shc Family of Adaptor Proteins

**DOI:** 10.5334/1750-2187-12-2

**Published:** 2017-05-03

**Authors:** Samrein B. M. Ahmed, Sally A. Prigent

**Affiliations:** 1 Basic science department, college of medicine and Shariah institute for medical research, University of Sharjah; 2 Department of Molecular and Cell Biology, University of Leicester, UK

**Keywords:** Shc, Functional diversity, Adaptor proteins, Oxidative stress, Shc regulation

## Abstract

The Shc family of adaptor proteins is a group of proteins that lacks intrinsic enzymatic activity. Instead, Shc proteins possess various domains that allow them to recruit different signalling molecules. Shc proteins help to transduce an extracellular signal into an intracellular signal, which is then translated into a biological response. The Shc family of adaptor proteins share the same structural topography, CH2-PTB-CH1-SH2, which is more than an isoform of Shc family proteins; this structure, which includes multiple domains, allows for the posttranslational modification of Shc proteins and increases the functional diversity of Shc proteins. The deregulation of Shc proteins has been linked to different disease conditions, including cancer and Alzheimer’s, which indicates their key roles in cellular functions. Accordingly, a question might arise as to whether Shc proteins could be targeted therapeutically to correct their disturbance. To answer this question, thorough knowledge must be acquired; herein, we aim to shed light on the Shc family of adaptor proteins to understand their intracellular role in normal and disease states, which later might be applied to connote mechanisms to reverse the disease state.

## Background

Signal transduction is essential for translating extracellular events into appropriate biological responses. For this reason, intracellular networks need to be tightly regulated. Adaptor proteins are one of the factors that maintain intracellular homeostasis; any disturbance in the regulation of adaptor proteins may lead to a disease condition or, in some cases, may trigger cancer formation. One of the most studied adaptor protein families is the Src homology and Collagen (Shc) family, which is implicated in both physiological and disease conditions. The Shc family of docking proteins is an essential element in signalling cascades mediated by different extracellular signals, including growth factors, cytokines and integrins [1]. Shc proteins generally exert their action by activating mitogen-activated protein kinases (MAPK) and phosphoinositide-3-kinase/Akt signalling pathways [1,2,3].

**Figure 1:**
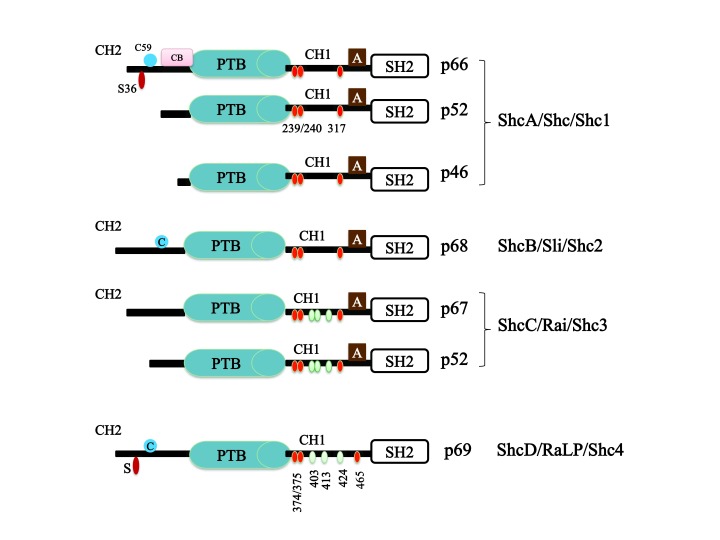
**Schematic representation of the structural modularity of the Shc family of proteins.** Shc proteins share the same structural hallmarks of highly conserved PTB and SH2 domains and poorly conserved CH2 and CH1 domains. Three conserved tyrosine phosphorylation sites exist in the CH1 domain (yellow). In ShcC and ShcD/RaLP, additional tyrosine phosphorylation residues (non-conserved) are present in the same CH1 domain (green). SER36 in the p66ShcA-CH2 domain is responsible for the oxidative-stress response function. RaLP-CH2 also contains a putative serine site for phosphorylation. A cysteine (C) residue in the p66ShcA-CH2 domain of ShcB and ShcD/RaLP (blue) is involved in oligomerization. A cytochrome c (CB) binding site is present only in p66ShcA but not in other Shc proteins (pink). An Adaptin binding motif (A) is present in all Shc proteins except ShcD/RaLP (cream-coloured) (adapted from Melanie and Jones, 2012).

Adaptor proteins facilitate protein-protein interactions, which are responsible for intracellular signal propagation as well as organization. The Shc family consists of a group of adaptor proteins that are evolutionary related by some of their shared functional and structural features. The distinctive structural feature of Shc proteins is that they contain an amino terminal phosphotyrosine binding (PTB) domain, a linker collagen homology 1 (CH1) domain and a carboxy terminal Src homology 2 (SH2) domain. An additional amino terminal collagen homology region (CH2) exists in the longest Shc protein transcripts [1,4,5,6] (Figure 1).

The Shc family consists of four members encoded by four distinct genes: Shc/Shc1/ShcA, Sli/Shc2/ShcB, Rai/Shc3/ShcC and RaLP/Shc4/ShcD [6] (Figure 1). Broadly, Shc proteins contribute to mediating several intracellular signalling cascades; they are implicated in cell proliferation, cell differentiation, cell survival and migration [7,8,9]. In addition, their roles in angiogenesis and tumourigenesis have been determined [6,10]. ShcA is the most studied member because it is widely expressed in human tissue [11].

## Functional diversity of the Shc family

Shc family members mediate signal transduction to trigger different biological events. Many factors contribute to the functional diversity of Shc proteins: their gene products can produce more than one transcript, which each have a particular role; multiple domains offer Shc proteins the ability to bind to different signalling proteins; Shc proteins possess various serine, threonine and tyrosine residues that can potentially be phosphorylated; and the structural properties of Shc proteins facilitate their relocation to different intracellular organelles. All the above factors grant Shc family members the capacity to regulate various signalling cascades in the different intracellular compartments.

### The expression profiles of Shc proteins and their isoforms

The Shc family consists of at least seven identified proteins arising from four different genes. This is attributed to the alternative use of the initiation codons and mRNA splicing [12]. The presence of the Shc protein isoforms provides the Shc family with a range of functions. Shc scaffolds show distinct expression patterns that facilitate the propagation of messages in different cellular settings.

The ShcA protein is present in the intracellular compartment in three isoforms, p46, p52 and p66ShcA (Figure 1), which are each implicated in certain intracellular pathways. The three isoforms arise from two mRNAs derived from the Shc gene, which encodes p66ShcA and p46/p52ShcA [1,13].

Although all ShcA isoforms are derived from the same gene, p66ShcA has an extension of 110 amino acids at its amino-terminus. This extension has provided p66ShcA with a distinct role from the two shorter isoforms (p46ShcA and p52ShcA). p46ShcA and p52ShcA are involved in cell cycle progression and cell differentiation [14,15], whereas p66ShcA plays a role in apoptosis in response to oxidative stress [16]. Unlike the shorter Shc isoforms, p66ShcA prevents Ras activation, resulting in the suppression of lung cancer cell motility [17]. Another difference between the expression profiles of ShA isoforms expression has been reported; in contrast to p46 and p52 ShcA, p66ShcA expression is absent in human haemopoietic cells [11].

The ShcA protein was shown to be ubiquitously expressed in all tissues except the adult mature brain. Unlike ShcA, ShcB, ShcC and ShcD are abundantly expressed in the brain [12,18]. ShcB and ShcC, with its two isoforms (p67, p52), were determined to be involved in neuronal cell development and survival, while the main function of ShcB is linked to the peripheral nervous system rather than the central nervous system [19] (Figure 1). The dominance of ShcC function in brain signalling originated from the fact that ShcC has a higher affinity for nerve growth factor (NGF) and brain derived-neurotrophic factor (BDNF) receptors compared to ShcB [18]. Unlike ShcC, ShcB is present in retina, heart and vascular endothelial cells [20], while ShcC shows a unique expression in lymphocytes that is not shown by ShcB [21].

Recently, the new Shc family member RaLP/ShcD was shown to be expressed in the brain, neuronal-derived tissues (e.g., melanocytes), and skeletal muscle in adult mice. RaLP/ShcD is reported to trigger melanoma invasion and maturation of the neuromuscular junction, and it has been shown to contribute to mouse embryonic development [6,12,22]. Despite the reported intracellular roles for RaLP, the exact molecular mechanisms by which RaLP achieves these functions are still unclear.

An interesting example of the switch in expression between Shc adaptor proteins to satisfy cellular needs was described by Conti et al. This was demonstrated by implementing two-colour fluorescence-activated cell sorting, which enabled the researchers to show that ShcC expression is limited to the post-mitotic neuronal cells while ShcA is mainly available in neuronal progenitors. ShcA expression is initially required to facilitate progenitor cell proliferation and is then replaced by ShcC, which assists in neuronal cell maturation by mediating cell differentiation and survival. Accordingly, it was suggested that modifying ShcA and ShcC expression during the development of the brain would act as a crucial event for determining the proliferation and differentiation of neuronal cells [9].

### Shc protein domains

Although Shc adaptor proteins lack intrinsic enzymatic activities, they are equipped with different domains (CH2, PTB, CH1 and SH2) that are defined by distinctive amino acids motifs and sequences that help the Shc proteins to convey, organize and amplify the signals received.

#### Phosphotyrosine binding domains

Shc proteins are the only adaptor proteins that possess both a PTB (phosphor-tyrosine binding) domain as well as a SH2 (Src homology 2) domain [1]. PTB and SH2 domains are the most conserved regions among Shc family members [6], and they bind to phosphor-tyrosine motifs, allowing Shc proteins to be recruited to activated receptors, including receptor tyrosine kinases (RTKs), antigen receptors, G-protein coupled receptors and cytokine receptors [3,5,23,23,24,25]. The Shc phosphotyrosine binding domains also enable Shc proteins to interact with cytosolic tyrosine kinases [26]. The Shc-PTB domain binds the phosphorylated tyrosine present in the NPXpY motif, while the SH2 domain preferentially interacts with a phosphorylated tyrosine contained in a pYXXФ motif (where Ф represents a hydrophobic amino acid) [1].

The combination of SH2 and PTB domains enables Shc to switch between signalling pathways; it was shown that the SH2 domain is specifically needed in cell proliferation, while the PTB domain is important in cell migration [27].

Site-directed mutagenesis revealed that R175 and R285 in the tyrosine binding pockets of the PTB domains in p52Shc and p66ShcA, respectively, are pivotal for anchoring Shc to certain tyrosine phosphorylated proteins [23,28].

In addition to the role of the Shc-PTB domain in binding phosphotyrosine residues, the PTB domain also contributes to localizing Shc to the cytosolic side of the plasma membrane via its interaction with plasma membrane phospholipids [25]. NMR studies have shown that the residues responsible for binding the membranous phospholipids are distinct from the phosphotyrosine binding residues [29]. This implies that recruitment of Shc to the inner membrane does not always require activation of the upstream receptors. Notably, the mutation of Met46 to Pro in the p52ShcA-PTB domain abolished Shc’s ability to activate c-Src, which subsequently diminished Stat-induced p21/cip upregulation [30].

The PTB domain of p52ShcA contains a potential phosphorylated Ser29 residue in the RHGSFVNK motif that has a role in the interaction between Shc and the tyrosine phosphatase PTP-PEST, which acts as a safety valve to hinder the flow of ligand-induced signalling via its association with ShcA [31].

Although tyrosine phosphorylation is a requirement for the coupling of the PTB and SH2 domains in Shc with other proteins, it was reported that the PTB domain binds PTP-PEST and IQGAP independently of tyrosine phosphorylation [31,32]. Additionally, ShcA associates with Ran-GTPase via the SH2 domain of ShcA; this interaction does not require tyrosine phosphorylation of ShcA [33]. As a consequence, the Shc-SH2 domain initiates Shc nuclear translocation without binding to tyrosine phosphorylated sites.

#### Proline and Glycine rich domains

The collagen homology regions include the CH1 and CH2 domains. The CH1 domain links the PTB and SH2 regions while the CH2 is located at the extreme N-terminal region of the Shc proteins. These domains were designated by these names (CH1 and CH2) because they share 50% homology with human α1 collagen, which is rich in glycine and proline. The collagen homology regions of Shc proteins also possess a frequently occurring conserved GXX motif, where X is generally lysine or proline [11]. Importantly, the CH2 domain does not exist in the shorter isoforms of Shc family members and is only present in p66ShcA, p68ShcB, p67ShcC and p69ShcD/RaLP. The proline and glycine stretches enable the collagen homology domains to act as docking sites for proteins containing SH3 domains [1]. Both the CH1 and CH2 domains represent the least conserved regions among Shc family members [12].

Shc proteins can be phosphorylated by tyrosine kinases on certain conserved tyrosine residues contained within the CH1 region [27] (Figure 1). The phosphorylated tyrosine residues in the CH1 domain represent binding sites for many proteins, the best characterized of which is Grb2. Grb2 recruits Sos, which activates the Ras/MAPK signalling pathway [34]. ShcC and ShcD/RaLP were reported to have additional potential tyrosine phosphorylation sites in the CH1 domain [12], which might contribute to the activation of unique signal transduction pathways (Figure 1).

The ShcA-CH1 region also contains the Adaptin interacting motif (amino acids 346–355), which contributes to the ability of Shc proteins to associate with endosomes [35]. Unlike the CH1 domain, the CH2 region is likely to be phosphorylated on serine and threonine residues. One of the most studied examples is serine 36 in p66ShcA-CH2, which plays a pronounced role in the oxidative-stress response mediated by p66ShcA [16] (Figure 1). p66ShcA also houses a cytochrome c interacting region at the carboxy-terminal of the CH2 domain [36] (Figure 1). In addition, the p66ShcA-CH2 domain contains cysteine 59, which is responsible for protein dimerization (Figure 1) and has been suggested to have an effect on the p66ShcA-mediated stress response [37]. An interesting observation was made by Khanday et al. regarding the inhibitory effect of the p66ShcA-CH2 domain on p66ShcA/Ras signalling. Under oxidative stress conditions, the CH2 domain of p66ShcA was found to dissociate Sos1 from Grb2-SH3; this dissociation mediates the association between Sos1 and eps8, which in turn leads to the activation of Rac1 instead of Ras [38]. Furthermore, cytoskeletal reorganization was reported to mediate Erk activation via the phosphorylation of p52ShcA or p46Shc but not p66ShcA. This was attributed to the p66ShcA-CH2 sequence having an inhibitory effect on cytoskeletal reorganization-mediated phosphorylation [39]. In a recent paper, the CH2 domain of ShcD was shown to house a nuclear export signal that might play a role in ShcD nuclear cytoplasmic shuttling [40].

### Shc protein localization

The extracellular events influence the internal environment of the cell to elicit specific responses. Certain proteins sense the extracellular signals and transmit them to mediate relevant intracellular cascades. Shc members assist in transmitting signals through different ways, such as by inducing relocation into different subcellular compartments. As Shc proteins were originally characterized as adaptors coupling receptor tyrosine kinases to downstream signalling pathways, it was thought that Shc members are cytoplasmic proteins. However, recent reports have shown the presence of Shc proteins in different intracellular compartments.

p52ShcA and p46ShcA are mainly distributed in the cytoplasm. However, upon their tyrosine-phosphorylation, they translocate to the membrane and convey the extracellular signals to the intracellular environment [41]. In addition, the Shc-PTB domain contributes to the membrane localization of Shc [29]. Because p66ShcA plays a role in reactive oxygen species metabolism, its mitochondrial translocation has been analysed [42].

The p46ShcA isoform was also shown to translocate into the mitochondrial matrix by means of a mitochondrial target sequence within its PTB domain [43].

Interestingly, the nuclear localization of p46Shc was reported in both hepatocytes and hepatic carcinoma cells [44]. Because Shc isoforms lack the classical nuclear localization signal (NLS), it was thought they translocate to the nucleus through a NLS-independent mechanism. Only the shorter isoforms (p46ShcA and p52ShcA) of ShcA were shown to co-immunoprecipitate with the overexpressed Ran-GTPase, a key component of the nuclear transport machinery. The association of ShcA/Ran was enhanced by serum stimulation after the cells were deprived from growth factors [33]. Using FRET, researchers clearly showed a direct interaction between the two proteins in the nucleus, and it was suggested that Ran mediates Shc nuclear import. The fact that Ran is upregulated in cancer cells led to the assumption that Shc nuclear translocation might contribute to the oncogenic effects of Ran. The interactome data in that study indicated that some nuclear proteins might associate with Shc, highlighting its role in the nucleus [45]. Additionally, the newly identified ShcD was found to shuttle between the nucleus and the cytoplasm upon oxidative stress [40].

Subcellular fractionation revealed the presence of tyrosine phosphorylated Shc, Grb2, SOS and active EGFR in the endosomes in response to EGF treatment [46]. In unstimulated cells, Shc was shown to reside on the rough endoplasmic reticulum mainly within the perinuclear cisternae, while treatment with EGF stimulated the redistribution of Shc both to the membrane region and to endosomes [47]. AP2 has a well-studied role in clathrin-dependent endocytosis [48]. In cells subjected to epidermal growth factor (EGF), Shc interacts with AP2, which mediates EGFR internalization by early endosomes [35,49,50].

NGF treatment prompted ShcA translocation to the cytoskeleton and the membranous regions and triggered the ShcA-actin interaction [51]. The association of ShcA with the cytoskeleton was later confirmed by Smith et al. In epidermal growth factor (EGF)-treated metastatic bladder cancer cells and a skin carcinoma cell line, immunostaining for Shc and the cytoskeleton-associated protein IQ Motif Containing GTPase Activating Protein 1 (IQGAP1) demonstrated the recruitment of the two proteins to the membrane ruffles and to the lamellipodia. Depleted levels of ShcA negatively affect the formation of motility structures and result in failure of IQGAP1 recruitment to the membranous region and to lamellipodia [32]. Alternatively, protein kinase C-δ (PKCδ) was found to regulate Shc translocation to the cytoskeleton and mitochondria in H_2_O_2_-treated cardiomyocytes [52].

Interestingly, the stimulation of α_6_ β_4_ integrin in the presence of laminin resulted in p52ShcA recruitment that led to phosphorylation of p52ShcA [53]. Hemidesmosomes are considered a key element in epithelial cell-basement membrane adhesion [54]; α_6_ β_4_ integrin is a component of hemidesmosomes [55]. Accordingly, these data indicated a new ShcA subcellular localization in the hemidesmosome territory, which may suggest a function for ShcA in cell-basement membrane adhesion.

Shc adaptor proteins respond to various stimuli by altering their intracellular distribution to relay messages, activating different downstream pathways dictated by the cellular needs.

## The role of the Shc family in different intracellular signalling cascades

**Figure 2:**
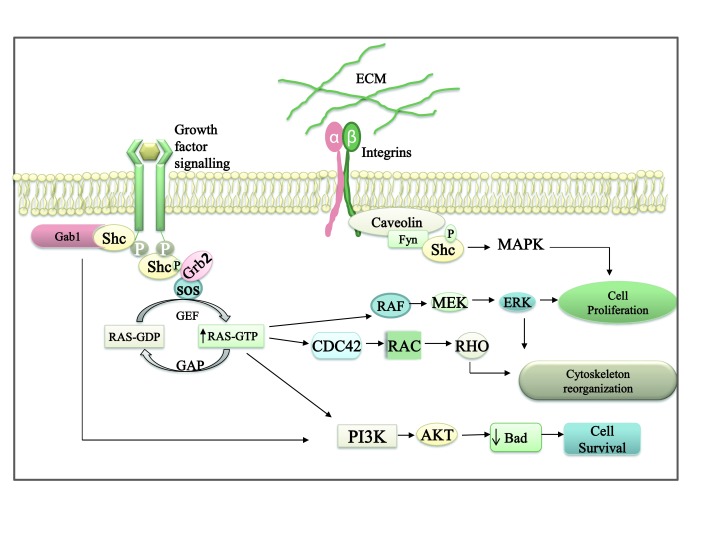
**Shc acts as a bridge connecting the extracellular signal and the different intracellular signalling pathways.** Shc is recruited by activated cell surface receptors or integrins. Shc can then be phosphorylated either by receptor **tyrosine kinases** (EGFR) or cytosolic **tyrosine kinases** (Fyn, a protein of the Src family). Phosphorylated Shc can then interact with Grb2, leading to the activation of different pathways such as MAPK, PI3K/Akt and RhoA via the activation of Ras. Therefore, Shc proteins trigger different intracellular cascades, resulting in various cellular responses (adapted from Zhu and Parada, 2002).

The fact that the Shc family comprises different proteins provides functional diversity to this family. Shc proteins have been shown to be involved in cell proliferation, differentiation, migration, the oxidative stress response and survival (Figure 2). It was originally thought that the actions of Shc proteins were restricted to transducing signals from upstream receptors, such as RTKs, cytokine receptors and integrin, though many recent reports have revealed different aspects of Shc signalling.

### Shc and cell proliferation

In mouse fibroblasts, the activated EGF receptor interacts with ShcA, resulting in ShcA phosphorylation on tyrosine 239/240 and 317; in turn, phosphorylated ShcA binds growth factor receptor-bound protein 2 (Grb2) (Figure 2). The Shc/Grb2 complex recruits Son of sevenless (Sos), a guanine nucleotide exchange factor of Ras. The activation of Ras induces the Raf/MAPK pathway, which subsequently leads to the upregulation of c-fos that drives mitogenesis [27] (Figure 2).

The role of Shc in cell proliferation is not confined to MAPK activation; ShcA can exert its mitogenic effect by inducing c-Myc expression [26]. In response to EGF stimulation, the phosphorylation of ShcA at tyrosine residues 239 and 240 has been shown to induce c-Myc-mediated cell proliferation [56]. Upon the stimulation of pre-T cell receptors in undifferentiated thymocytes, ShcA displayed an important role in sorting a mitogenic signal via c-Myc activation [57]. Moreover, in primary fibroblasts cells, ShcA was found to play a role in integrin (α5β1, α1β1 and αvβ3)-mediated proliferation [58] (Figure 2).

Conversely, the dephosphorylation of ShcA via its binding to SHIP phosphatase or PTP-PEST is implicated in Ras/MAPK downregulation, which was shown to adversely affect the mitogenic signal in haemopoietic cell lines [31,59].

### The role of Shc in cell survival and oxidative stress

#### The impact of Shc on mediating the survival signal

On the basis of signalling pathway crosstalk, Shc can activate the PI3K/Akt pathway via the MAPK pathway and can thereby promote cell survival [1,3] (Figure 2). In addition, Shc was reported to interact with Grb2-associated binding protein 1 (Gab1), which is known to activate the PI3K/Akt pathway [60]. Furthermore, the role of ShcB and ShcC in promoting cell survival through inducing the PI3K/Akt pathway in certain subsets of neurons has been discussed by Skai and co-workers [19].

However, upon cell exposure to oxidative stress, p66ShcA becomes phosphorylated at the Ser36 residue, leading to the activation of a p53-independent pathway which, in turn, induces apoptosis [16].

ShcC, which is upregulated by high grade astrocytomas, was found to be phosphorylated at tyrosine residues by Ret tyrosine kinase receptor. The ShcC/Ret association was revealed to play a role in astrocytoma survival through triggering the PI3K/Akt signalling pathway [8]. In a different study, ShcC was elucidated to mediate prosurvival signals by both Ret-dependent and independent pathways, as a relative increase in the phospho-Akt levels were observed downstream of unstimulated and stimulated Ret receptors. In addition, the overexpression of ShcC provided PC12 cells with resistance to the death signal generated by serum starvation [61]. Surprisingly, in contrast to its the anti-apoptotic functions in neurons, ShcC was shown to impair the survival signal of T and B lymphocytes [21].

Parallel to the role of p66ShcA in oxidative stress-induced apoptosis, p66ShcA was found to initiate anoikis and apoptosis due to the loss of contact between integrin and the exterior of the cell [62] when the cells were forced to grow in suspension [63]. In contrast to the impact of p66ShcA in oxidative stress-induced apoptosis [64], ShcC plays a role in protecting neuronal cells from death following oxidation or hypoxic insults via the induction of the PI3K/Akt signalling cascade [65]. In haemopoietic cells, ShcA induces a survival signal in early developing B cells that facilitates B cell development [66]. An interesting report showed that betulinic acid mediates ROS elevation, which ultimately results in the apoptosis of hepatocellular carcinoma cells via the upregulation of p66Shc and miR-21 [67].

#### Shc as a key regulator in promoting the oxidative stress response

Shc proteins are commonly involved in transmitting signals from upstream receptors to downstream effectors. p66ShcA has shown an interesting role in mediating the stress response. p66ShcA-/- knockout mice displayed a 30% increase in life span and less susceptibility to oxidative stress compared to their wild type counterparts [16], which highlighted a new role for Shc in the stress response and ageing. In the same study, Migliaccio et al first demonstrated the contribution of p66ShcA to signal transduction pathways involved in the response to reactive oxygen species (ROS). That study also exposed the crucial role of Ser36 phosphorylation of p66ShcA in the induction of the oxidative stress response. Interestingly, p66ShcA was revealed to upregulate p21/cip levels in response to oxidative stress in a mechanism that is suggested to be independent of p53 [16].

**Figure 3:**
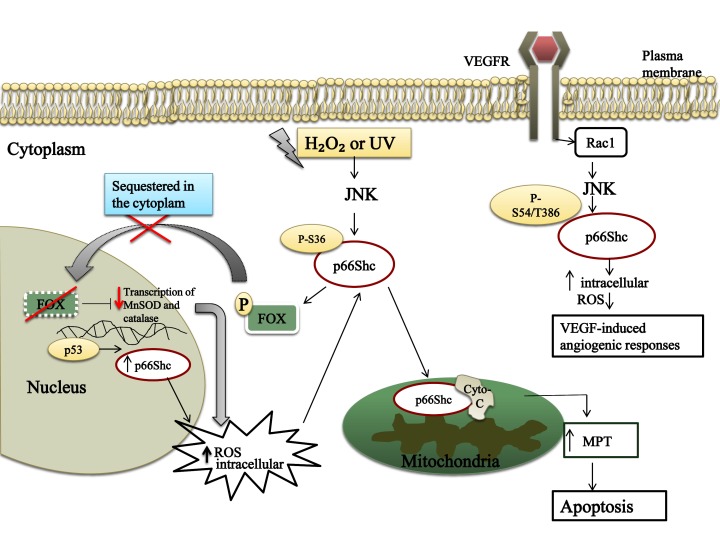
**p66ShcA has a key role in the oxidative stress response.** p66ShcA is phosphorylated on Ser36 via the activation of stress **kinases** such as JNK after the exposure of cells to oxidative stress agents (e.g., H_2_O_2_ and UV). This results in translocation of a fraction of p66ShcA into the mitochondrial intermembrane space, where it binds physically with cytochrome c (Cyto-c), which results in a mitochondrial permeability transition (MPT). The release of cytochrome c results in apoptosome formation, initiating the apoptotic process. p66ShcA mediates the transcription factor FOX phosphorylation, which results in an inhibition in the transcription of scavenging enzymes such as catalase and MnSOD. Upon VEGF stimulation in endothelial cells, p66ShcA is phosphorylated at Ser54 and Thr286 in response to Rac1 activation. Consequentially, the produced intracellular reactive oxygen species (ROS) initiate the VEGF-mediated angiogenic response.

p66ShcA was also revealed to contribute to neuronal death in Alzheimer’s disease in response to amyloid β (Aβ)-peptide accumulation, which results in oxidative stress. p66ShcA influences the production of ROS in the intracellular compartment and particularly in the mitochondria, which induces Aβ-mediated cell death. This function of p66ShcA is clearly related to its phosphorylation at Ser36, which is partially dependent on JNK activation [68]. In the same study, p66ShcA phosphorylation was found to elevate ROS levels by negatively regulating the transcription of ROS-scavenging enzymes, such as Mn superoxide dismutase (MnSOD) and catalase, mainly by intervening with phosphorylated forkhead proteins [68,69] (Figure 3). Strikingly, p66ShcA was reported to act as one of the downstream effectors of p53, and the upregulation of p66ShcA by p53 was observed upon UV treatment (Figure 3). Thorough experiments have shown that p66ShcA has a key role in p53-mediated ROS elevation and apoptosis [70].

In a different context, constitutively active Rac1 induces ROS production partially by mediating p66ShcA phosphorylation at Ser54 and Thr386 via the p38MAPK and JNK stress kinases. The phosphorylation of p66ShcA protects the protein from degradation by reducing its ubiquitination. This study provided a new mechanism for regulating the ShcA protein [38]. Likewise, the association of p66ShcA and Rac1, which was induced upon vascular endothelial growth factor (VEGFR) activation, was investigated in endothelial cells. The interaction was found to induce ROS production that triggers capillary branch formation, suggesting that p66ShcA contributes to VEGF-mediated angiogenic responses in endothelial cells [10] (Figure 3). Indeed, p66ShcA has been shown to be a key regulator in oxidative stress conditions; these events do not always result in cell death, but they might also yield different responses that are linked to angiogenic-linked diseases such as atherosclerosis and diabetes [71,72].

Some experimental evidence demonstrated a role for p66ShcA in oxidative-stress induced apoptosis, but the exact mechanism was not clear until a study was conducted by Orsini et al. The treatment of mouse embryo fibroblast cells (MEFs) with staurosporine, a proapoptotic agent that induces the release of cytochrome c and the activation of caspase-3, resulted in diminished levels of cleaved caspase-3 in p66ShcA-/- cells when compared with wild type MEFs. The proapoptotic function of p66ShcA was proposed to result from changes in the mitochondrial transmembrane potential, leading to the release of cytochrome c into the cytoplasm which, in turn, results in apoptosis. The apoptotic role of p66ShcA was shown to be repressed by mitochondrial HSP70, which forms a complex with p66ShcA in non-oxidative stress conditions [73].

Liver cells obtained from wild type (wt) and p66ShcA knockout mice were treated with CCl4, a chemical responsible for ROS generation. The mitochondria were then isolated and tested for the levels of ROS by spectrofluorimetry. The wild type cells exhibited an elevation in ROS level proportional to the increase in the fluorescence of 2’,7’-dichlorofluorescin diacetate (H_2_DCFDA), a compound that fluoresces in response to oxidation by ROS; by contrast, ROS levels persisted unaltered in the p66ShcA-/- cells [74]. Further studies were conducted to reveal the mechanism underlying the role of p66ShcA in the production of mitochondrial ROS. p66ShcA was found to interact physically with the reduced cytochrome c, which led to cytochrome c oxidation and ROS production [74] (Figure 3).

A novel mechanism describing how p66ShcA regulates cellular ROS was illustrated by Kumar et al. In endothelial cells, p66ShcA potentiates ROS production by constraining the expression of Kruppel-like factor 2 (KLF2), which was found to induce the expression of catalase, an ROS scavenging enzyme [75,76]. In UV and H_2_O_2_ treated melanoma cells, the function of p66ShcA in inducing ROS production was found to be abolished by the secreted protein, melanoma inhibitory activity (MIA) [77].

p66ShcA ablation in MEFs resulted in less ATP generation and reduced oxygen consumption by the mitochondria, which consequently resulted in a decline in ROS levels. By contrast, an elevated level of lactate was observed.

p66ShcA-/- cells meet their energy requirements by using an alternative pathway, which generates ATP by converting pyruvate into lactate. The loss of p66ShcA in these cells also reduced the degree of regeneration of NADH from NAD+. This study provided mechanistic evidence of the role of p66ShcA in regulating the intracellular reactive oxygen species as well as in the ageing process [42]. Interestingly, p66ShcA acts as a downstream effector for p53-induced apoptosis following oxidative stress [70]. In contrast, Apurinic/apyrimidinic endonuclease 1 (APE-1), a multifunctional protein that plays a role in DNA repair and in regulating intracellular ROS [78], dephosphorylates p66ShcA by inhibiting PKC [79].

#### The role of the Shc family in cellular migration and metastasis

In addition to their roles in cell proliferation and survival, Shc proteins are also implicated in cell motility. Shc acts as a regulatory molecule in directing intracellular pathways to promote either proliferation or migration through the alternative use of the phospho-tyrosine binding domains (PTB and SH2). The PTB domain has been shown to drive the cell towards haptotactic (directional) movement, while the SH2 domain was found to be involved in cell cycle progression [27].

PTEN is known to be a negative regulator of the Akt pathway, which is involved in cell survival, by converting PIP3 to PIP2. It was reported that 35% of melanoma cell lines downregulate PTEN. Interestingly, a novel function of PTEN was found in which PTEN downregulates focal adhesion kinase (FAK) and ShcA by directly dephosphorylating their modified active forms. Based on this finding, when PTEN is depleted, ShcA and FAK actively promote tumour development, migration and invasiveness [80,81]. Schneider et al 2010 analysed the role of PTEN in the migration of renal cancer cells and found that PTEN expression is related to fewer metastatic tumours due to the de-phosphorylation of ShcA, while FAK phosphorylation remained unchanged [82].

Moreover, ShcD/RaLP has been found to be overexpressed in invasive melanoma, inducing both Ras-dependent and independent migratory pathways [6]. p66ShcA was found to be upregulated in breast cancer accompanied by Ras-independent nodal invasion, thereby suggesting a role for ShcA in breast cancer metastasis [83].

In a different study, mutant forms of the Met oncoprotein, which associates only with PLCγ, the p85 subunit of PI3K, Grb2 and ShcA, were generated. This enabled the contribution of individual pathways downstream of Met receptor tyrosine kinase to be evaluated. Unlike other tested adaptor proteins (PLCγ and p85 subunit of PI3K), ShcA and Grb2 were able to induce lung metastases in nude mice [84,85]. Additional work by Hudson et al confirmed the downstream role of p66ShcA in the Met-induced epithelial mesenchymal transition in (HER2 +) luminal breast cancer cells [86].

In contrast to studies implicating various Shc proteins in cell migration, p66ShcA overexpression in Lewis lung carcinoma provided the cells with striking resistance to migration. This result was due to the inhibiting effect of p66ShcA on Ras activation [17]. Furthermore, p46/p52ShcA interacts with α5β1 integrin, the fibronectin (FN) receptor, and significantly modulates the association of the extracellular matrix with integrin that leads to an accelerated spread of MCF-7 breast epithelial cells on FN. The ectopic expression of p52ShcA in MCF-7 cells augmented the rate of adhesion to fibronectin, thereby negatively affecting cell motility. Interestingly, the use of FN-coated inserts in Boyden chambers to explore the role of ShcA revealed that ShcA expression has an impact on promoting cell motility, in particular upon treatment with EGF or IGF-1 [87]. The association of ShcA with α5β1 has revealed another aspect of Shc-mediated regulation of cell motility and adhesion.

In breast tumour cells expressing activated ErbB-2, treatment with TGF-β results in a striking increase in cell motility compared to cells expressing mutant inactive ErbB-2. This finding indicated the possibility of synergism between the ErbB-2 and TGF-β pathways. The Shc protein was shown to play a central role in the cooperation between the ErbB-2 and TGF-β pathways. In the same cell system, ShcA expression was shown to be crucial for TGF-β-induced focal adhesion turnover, which is indispensable for cell motility. Dominant negative p52ShcA (ShcA/3F), which lacks the three tyrosine residues (Y239/240 and Y317) essential for ShcA signalling, impaired TGF-β-mediated cell migration and invasion; this effect was found to be independent of Shc-Grb2 association [88].

#### Shc as a key element in cell differentiation

For cells to gain a specialized function, some molecular modifications should occur to facilitate the differentiation process. Shc proteins are needed for the specialization of particular types of cells, such as haemopoietic or neuronal cells.

In a haemopoietic cell line, the adaptor protein Shc acts as a mediator of differentiation induced by the cytokine receptor c-Mpl in response to thrombopoietin (TPO). Although the phosphorylation of tyrosine in ShcA was a consequence of this stimulation, it did not result in Ras/MAPK activation. The PTB domain of ShcA was found to be the key factor in this molecular event [89]. An additional implication for ShcA protein in haemopoiesis was revealed; ShcA tyrosine phosphorylation was found to be a crucial element for the transition of B cells from pre-pro B cells to pro-B cells, resulting in the formation of more mature and differentiated B cells [66].

Thomas et al described the role of ShcA in PC12 cells by testing the impact of the PTB and the SH2 domains of ShcA in transducing the signal downstream of TrkA, the NGF receptor. Upon stimulation of PC12 cells with NGF, ShcA was recruited to the activated TrkA receptor via its PTB domain, resulting in phosphorylation of ShcA on tyrosine 239/240. This interaction proved to be important for NGF-mediated neurite extension in PC12 cells [90]. The involvement of ShcA in PC12 differentiation was studied further by Hinsby et al. In a co-culture method, PC12 cells were seeded on a monolayer of fibroblasts that either expressed or did not express the neuronal cell adhesion molecule (NCAM). NCAM was found to form a complex with the focal adhesion kinase (FAK) and Fyn tyrosine kinase, which resulted in ShcA phosphorylation. The association of phosphorylated ShcA and Grb2 was revealed to have a central role in NCAM-mediated neurite outgrowth [91].

Further insights into the roles of Shc proteins in differentiation indicated that the ectopic expression of ShcC in neural progenitors and in post-mitotic cells not only resulted in an increase in survival but also resulted in neurite elongation, which is a feature of neuronal differentiation via the PI3K-Akt pathway [9].

#### Shc proteins are involved in oncogenic events

Initially, ShcA protein oncogenicity was inferred by the ability of ShcA to transform mouse fibroblast cells. The ectopic expression of ShcA in NIH3T3 fibroblasts provided the cells with the ability to form colonies on soft agar and the ability to form tumours when injected into nude mice [11]. In contrast to the transforming function of ShcA in NIH3T3 cells, the overexpression of ShcA was not sufficient to give breast cancer cells a profound transforming advantage [87]. Tyrosine phosphorylation of ShcA also plays an important role in the morphological transformation of rat fibroblasts (Fr3T3] upon coupling with the Met oncoprotein receptor [84,85].

An in vivo experiment demonstrated the role of ShcA in ErbB2 mammary epithelial cell induced- tumourigenesis. The expression of ShcA in mammary epithelial cells is central to the ErbB2-mediated transition from hyperplastic cells to invasive cancerous cells [92].

Another interesting function of ShcA in mammary tumour progression is in prompting tumour angiogenesis. In mammary tumour cells expressing functionally active ErbB2, the overexpression of mutant ShcA (ShcA/3F), which lacks the three tyrosine residues (Y239/240 and Y317), strikingly affected the ability of ErbB2-transformed cells to secrete VEGF [92].

Interestingly, Middle T antigen (PymT), a component of polyomavirus, is responsible for the formation of haemangiomas, or blood vessel tumours that originate from the lining endothelial cells, in neonatal mice. A PymT mutant variant that was unable to recruit ShcA failed to transform endothelial cells. Subsequently, the PymT-mediated endothelial transformation phenomenon was found to occur via the formation of the Shc/Grb2/Gab1 complex, which mainly initiates the PI3K and Ras/MAPK pathways [93].

ShcC protein was found to be overexpressed and highly phosphorylated in high grade astrocytomas and in higher grade neuroblastomas [8,94]. Soft agar assays showed that ShcC mediates anchorage-independent growth in human neuroblastoma cells (TNB1). The oncogenic effect of ShcC in neuroblastoma cells was correlated partially to the formation of the ShcC/ALK complex, a process that is mainly dependent on the pronounced phosphorylation of ShcC [94].

Another in vivo study by Fagiani et al provided evidence for the role of Shc proteins in tumourigenesis. The injection of metastatic melanoma cells expressing high levels of RaLP into nude mice resulted in the formation of larger tumours compared with cells with depleted levels of RaLP [6].

#### Cardiovascular roles of Shc

The elimination of the three ShcA isoforms (p46Shc, p52Shc, and p66ShcA) was shown to produce embryonic lethality at day 11.5 due to cardiovascular defects [16]. A comprehensive study was conducted to rule out the signalling role of the PTB and SH2 domains of ShcA and the CH1 phospho-tyrosine sites in the developing heart. 48% of embryos that were deficient for functional Shc PTB died by day 11.5, while the remaining embryos experienced malformations in the cardiac trabeculae, enlarged hearts, and heart beat irregularity. In addition, the role of ShcA in ensuring the integrity of the developing heart was found to be independent of the SH2 domain and the CH1 phospho-tyrosine sites [95].

However, in Angiotensin (Ang) II-treated cardiomyocytes, the deletion of p66ShcA abolished Ang II-induced cardiac hypertrophy, which indicated the role of p66ShcA in G protein-coupled angiotensin receptor signalling [96].

The transmission of signals from tyrosine kinases by the PTB and SH2 domains of ShcA was found to be required for normal myocardial function. In the absence of p46ShcA and p52ShcA, but not p66ShcA, the myocardium experienced a disturbance in the extracellular matrix that was reflected by a disturbance in global heart contractility [97].

## Shc protein regulation

### Modulating Shc expression at the transcriptional level

Protein expression can be regulated at the transcriptional and translational levels or via the regulation of protein stability to elicit distinct responses and to cope with cellular needs. The mechanisms underlying Shc protein regulation have not been studied thoroughly; only a few papers describe the regulation of the longevity-associated protein p66ShcA.

Transfection of a p66ShcA promoter reporter construct into cell lines lacking endogenous p66ShcA expression resulted in normal promoter activity. This led to the suggestion that the promoter might possibly be regulated by epigenetic factors [98]. To further investigate this possibility, 32D haemopoietic cells without p66ShcA expression were treated with the histone deacetylase inhibitor (TSA) or with a demethylating agent (5-aza-dc), which resulted in p66ShcA expression at both the mRNA and protein levels. Histone deacetylases are usually recruited to methylation sites in a promoter [99]. Moreover, an analysis of the p66ShcA promoter in cell lines expressing high levels of p66ShcA revealed an unmethylated promoter [98]. Considering that histone deacetylases are implicated in the transcriptional repression of methylated promoters, this work indicates that p66ShcA expression is modulated at the transcriptional level by methylation [98].

In addition, elevated levels of homocysteine, an amino acid used in methionine biosynthesis that is linked to vascular endothelial dysfunction, have been proposed to result from the modulation of p66ShcA expression. Because homocysteine is involved in cellular methylation, it was hypothesized that homocysteine regulates p66ShcA promoter methylation, thereby modulating p66ShcA expression. Using bisulfite sequencing of genomic DNA from an endothelial cell line, methylated CPG dinucleotides were identified in the p66ShcA promoter; these dinucleotides were hypomethylated by homocysteine treatment. As a consequence of p66ShcA expression in response to homocysteine treatment, reactive oxygen species were elevated in endothelial cells [100].

### Shc proteins are post-translationally modified

#### Shc protein phosphorylation

The phosphorylation of Shc proteins plays a key role in the activation of intracellular cascades. Shc was shown to be phosphorylated at three tyrosine residues in the CH1 domain (Tyr317 and Tyr239/240) that were later shown to be conserved in the other Shc family members [1]. The tyrosine phosphorylation in the CH1 region is implicated in relaying the signal from activated tyrosine kinase receptors or cytoplasmic tyrosine kinases to the downstream effectors [26,101]. These events usually occur via Grb2 recruitment to the tyrosine phosphorylated ShcA and the association of these proteins with Sos leads to Ras/MAPK signalling pathway activation [102]. Parallel results showed that phosphorylated Y239/240 is involved in c-MYC induction rather than MAPK cascade activation [56].

**Figure 4:**
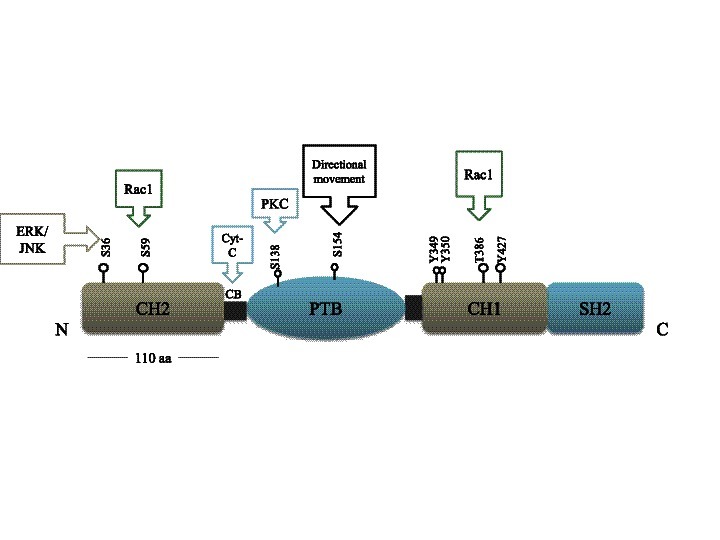
**Schematic representation illustrating the phosphorylation sites on p66ShcA.** There are three **tyrosine** phosphorylation sites and a threonine phosphorylation residue in the CH1 domain. There is one serine phosphorylation site on (Serine 138) the PTB domain as well as two serine phosphorylation sites in the amino terminal CH2 domain. p66ShcA has a unique cytochrome c binding region (CB). Ser36, Ser59, Ser138 and Thr386 are involved in the oxidative stress response, whereas S154 has a role in the directional movement of pancreatic cells. The phosphorylation of **tyrosine** residues in the CH1 domain are involved in MAPK activation (Adapted from Rajendran et al. 2010).

The Shc proteins contain different serine and threonine residues that can be phosphorylated. Phosphorylation of Ser36 in p66ShcA was found to have a role in the oxidative stress response [16]. Additionally, oxidative stress was reported to induce phosphorylation at Ser54 in the CH2 domain and at Thr386 in the CH1 domain of p66ShcA [38] (Figure 4).

Osmotic shock and insulin treatment induced the phosphorylation of serine in p66ShcA by MEK; similarly, serine and tyrosine become phosphorylated in p52ShcA in response to osmotic shock [103]. p66ShcA becomes serine/threonine phosphorylated upon EGF treatment, although unexpectedly, p66ShcA t impairs ERK activation in response to EGF stimulation [13]. Faisal and co-workers studied ShcA phosphorylation upon treatment with the PKC inducer 12-O-tetradecanoylphorbol-13-acetate (TPA). TPA treatment resulted in phosphorylation of Ser36, Ser138 and Thr29 in p66ShcA and phosphorylation of Ser29 in p52ShcA [31].

Additionally, the phosphorylation of Ser29 in p52ShcA and Ser138 in p66ShcA mediates the ShcA/PTP-PEST association; PTP-PEST is responsible for ShcA dephosphorylation following insulin stimulation [31]. Phosphorylation of p66ShcA at Ser36 is not only restricted to the oxidative stress response, it was also found to have a function in cardiomyocyte networks [104].

Unexpectedly, ShcC was found to be dephosphorylated on Ser/Thr residues subsequent to H_2_O_2_ or CoCl_2_ treatments (CoCl_2_ is a stimulator of hypoxia) [65]. Interestingly, unlike other reports that addressed the role of Shc serine and threonine residue phosphorylation in the stress response, Ser154 (in the PTB domain) was reported to be responsible for haptotactic movement of pancreatic cancer cells (Figure 4) [27].

In summary, serine/threonine phosphorylation of the ShcA protein is mediated by different stimuli, including H_2_O_2_, UV, Taxol, endothelin-1, Thrombin, norepinephrine, and PKC inducers such as TPA and PMA. Additionally, tyrosine phosphorylation of ShcA proteins is reported to be essential for transducing signals from the upstream tyrosine kinases to the downstream effectors.

#### Ubiquitination affects Shc protein stability

p66ShcA, which was found to be regulated at the protein level, is elevated in progressive types of prostate cancer, ovarian cancer, and breast cancer, which are steroid hormone-sensitive tumours [7]. It was therefore suggested that steroid hormones can affect p66ShcA expression. A 5α-dihydrotestosterone (DHT)-treated prostate cancer cell line and an oestrogen-treated ovarian cancer cell line showed elevated levels of p66ShcA when compared with untreated controls. Furthermore, treatment with MG132 (a proteasome inhibitor) caused increased levels of p66ShcA protein. The levels of deubiquitinated p66ShcA were clearly reduced after DHT treatment, which was similar to the effect induced by MG132 treatment [105]. It has previously been shown that the stability of p66ShcA is also induced by a p53-dependent mechanism under oxidative stress [70]; however, Kumar et al observed no alteration in p53 levels in their system [105].

P66ShcA phosphorylation on Ser54 and Thr386 by Rac1 under oxidative stress conditions also induces protein stability by deubiquitination [38].

#### A novel way Shc at the protein level

Remarkably, a recent report has described a ubiquitin-like modification that occurs on ShcA protein in T cells. Neddylation of ShcA by NEDD8 was shown to be essential for the recruitment of Grb2 and ZAP70, which are responsible for propagating signals involved in normal proliferation, cytokine production and differentiation via ERK activation [106]. Mass spectrometry revealed that neddylation occurs on lysine 3 of p52ShcA; mutating lysine 3 to arginine failed to elicit ERK activation. In contrast to the reported role of ubiquitin in Shc protein stabilization, the recent neddylation report has only defined the impact of Shc modification on protein interactions and subsequent signalling rather than protein stability [106].

## Conclusion

The most desirable therapeutic targets are cellular enzymes, such as tyrosine kinases and HDAC; however, many adaptor proteins, such as Shc proteins, IRS and Grb2, have been shown to be involved in different health disorders [107,108,109,110,111,112,113]. The use of highly potent and selective inhibitors to disrupt the binding abilities of bromodomains [114] has enabled the targeting of other adaptor proteins, including Shc proteins. Accordingly, this review shed light on Shc proteins to promote their consideration as possible therapeutic targets to improve the outcomes of Shc protein-mediated health disorders, such as ageing, cardiovascular problems, neurodegenerative diseases and cancer.

## Figures and Tables

**Table:** 

Akt	Protein kinase B
ALK	Anaplastic lymphoma kinase
AP2 complex	Assembly protein complex2
BDNF	Brain derived neurotrophic factor
CH	Collagen homology domain
C-terminus	Carboxy-terminus
EGF	Epidermal growth factor
EGFR	Epidermal growth factor receptor
ErBb2	Human epidermal growth factor receptor 2 (HER2/neu)
ERK	Extracellular signal-regulated kinase
FAK	Focal adhesion kinase
Gab1	Grb2-associated binding protein
Grb2	Growth factor receptor-bound protein2
H_2_O_2_	Hydrogen peroxide
IGF	Insulin growth factor
IGF1R	Insulin-like growth factor 1 receptor
IQGAP1	Ras GTPase-activating-like protein
IRS	Insulin receptor substrate
JNK	C-Jun N-terminal kinases
MAPK	Mitogen- activated kinase pathway
MEK	MAP/ERK kinase
Met	Mesenchymal-epithelial transition factor (HGF receptor)
MIF	Macrophage migration inhibitory factor
Mpl	Myeloproliferative leukaemia protein
NAD	Nicotinamide adenine dinucleotide
NADH	NAD+ hydrogen (H)
NCAM	Neural cell adhesion molecule
Nedd8	Neural precursor cell expressed developmentally down-regulated protein 8
N-terminal	Amino-terminal
PI3K	Phosphoinositide 3-kinase
PKC	Protein kinase C
PLCγ	Phospholipase C gamma
PC-PLC	Phosphatidylcholine-Selective phospholipase
PTB	Phosphorylated binding domain
PTEN	Phosphatase and tensin homolog
PTP-PEST	Protein tyrosine phosphatase contains PEST
RaLP	Rai-like protein
Ran	Ras-related nuclear protein
Ret	Rearranged during transfection
ROS	Reactive oxygen species
RTK	Receptor tyrosine kinase
Ser/S	Serine
SH2	Src homology 2
Shc	Src homology and collagen
SHIP	SH2 domain containing inositol 5-phosphatase
Sos	Son of sevenless
Src	Proto-oncogene tyrosine-protein kinase Src
TGF-β	Transforming growth factor-beta
Trk	Nerve growth factor receptor
Tyr/Y	Tyrosine
UV	Ultra-Violet light
VEGF	Vascular endothelial growth factor
